# Systemic lupus erythematosus complicated by severe Guillain-Barré syndrome: case report and literature review

**DOI:** 10.3389/fimmu.2025.1551448

**Published:** 2025-05-09

**Authors:** Lifeng He, Zengrui Zhang, Ying Tan

**Affiliations:** Department of Neurology, Huzhou Central Hospital, the Fifth School of Clinical Medicine of Zhejiang Chinese Medical University, Huzhou, Zhejiang, China

**Keywords:** case report, Guillain-Barré syndrome, systemic lupus erythematosus, peripheral neuropathy, acute progression stage

## Abstract

Systemic lupus erythematosus (SLE) is a heterogeneous chronic autoimmune disease characterized by immune-mediated multiple organ injuries in the setting of autoimmunity to nuclear antigens. In rare cases, it can complicated by the damage of peripheral nervous system, manifesting as Guillain–Barré syndrome (GBS). Severe GBS as the initial presentation is highly infrequent and associated with high disability and mortality rates, highlighting the importance of early detection, diagnosis, and treatment. Herein, we reported a successfully treated case of severe SLE-GBS in a 38-year-old male. Furthermore, we summarize the clinical characteristics of severe SLE-GBS reported thus far and propose the possibility of using medium-to-high dose corticosteroids in the acute progression stage of SLE-GBS. This report provides valuable insights for the analysis of disease characteristics and guidance for diagnosis and treatment of such cases.

## Introduction

Systemic lupus erythematosus (SLE) is a chronic diffuse connective tissue disease caused by abnormal activation of the immune system that attacks multiple systems throughout the body (1). When the peripheral nervous system is involved, it can manifest as Guillain–Barré syndrome (GBS) (1). GBS is an immune-mediated acute inflammatory peripheral neuropathy, with predisposing factors including infection, vaccination, and immune responses to other events (2). Severe GBS is characterized by rapidly progressive neurological deficits, notably ascending paralysis and bulbar involvement (dysphagia and dysarthria) which may culminate in respiratory failure. Concurrently, life-threatening autonomic instability—including malignant arrhythmias, labile hypertension, or hemodynamic collapse—further exacerbates clinical urgency, necessitating prompt recognition and immediate intervention to mitigate poor outcomes. Of particular clinical interest, while sporadic cases of SLE coexisting with GBS have been documented, the presentation of severe GBS as the inaugural manifestation remains an exceptional phenomenon. Herein, we report a successfully diagnosed and treated case of severe SLE-GBS and review relevant literature, summarizing the characteristics of such diseases to provide references for early identification and treatment optimization in the future.

## Case presentation

A 38-year-old male was admitted to the hospital following an 8-day history of progressive weakness and numbness in the limbs. The patient presented with malar rash but exhibited no symptoms, including fever, alopecia, photosensitivity, arthralgia, oral ulcers, dysphagia, diarrhea, or dysuria. Before admission, the patient had not received any treatment aimed at mitigating the disease. The patient’s medical history included hypertension, with no reported history of autoimmune or genetic disorders, and no relevant family medical history. Additionally, the patient denied any recent vaccinations or exposure to dog bites preceding the onset of neuromuscular symptoms.

**Figure 1:**
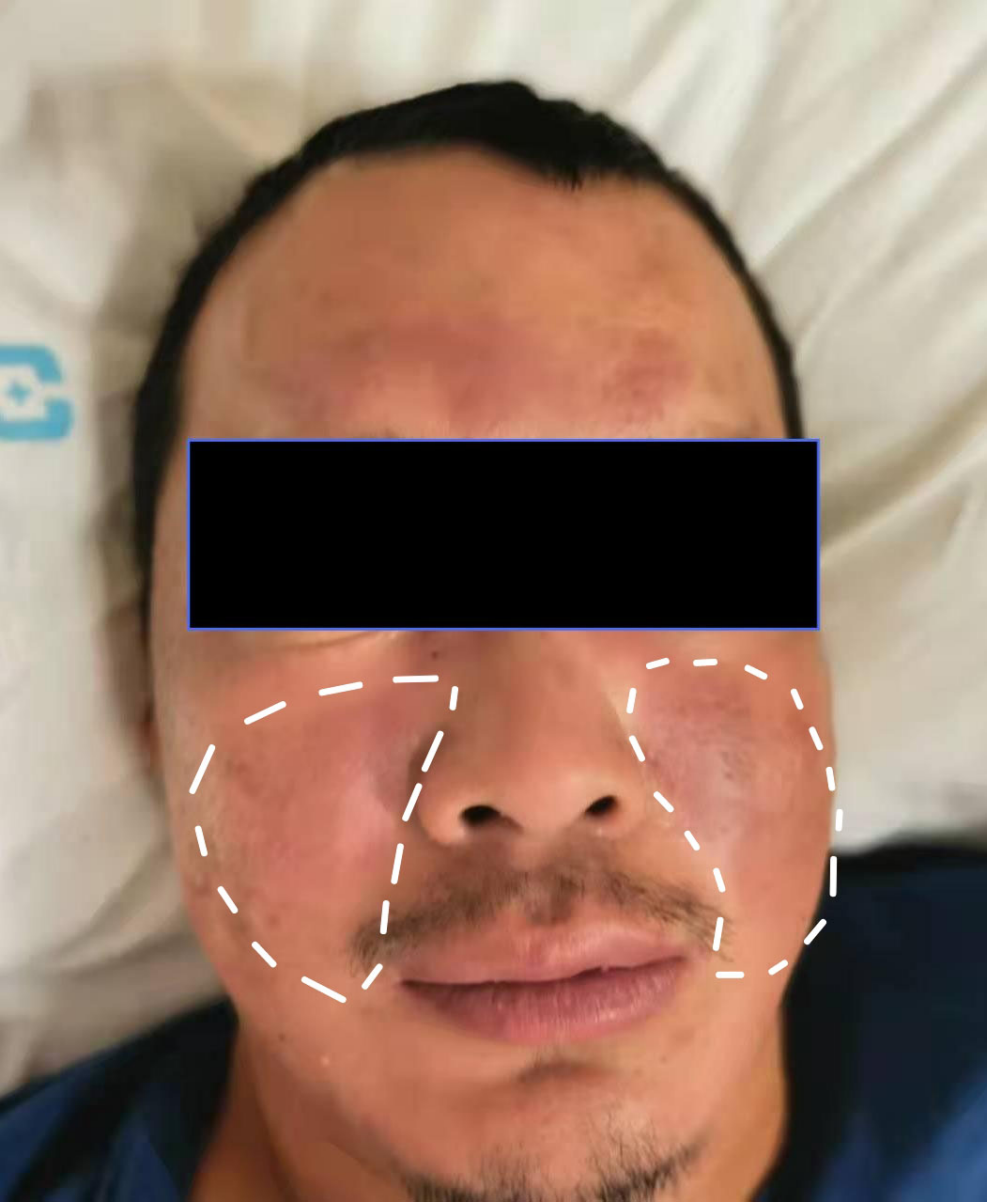
The malar rash on both sides of the face.

Upon physical examination at the time of admission, the only notable finding was a symmetrical butterfly-shaped erythema located in the zygomatic region (**Figure 1**). The vital signs were characterized by a maximum body temperature of 37.8°C, a blood pressure reading of 136/84 mmHg, a heart rate of 86 beats per minute, and a respiratory rate of 19 breaths per minute. Neurological assessment revealed a muscle strength of grade 3-/5 in the upper extremities and grade 2/5 in the lower extremities. Superficial sensation was diminished in the extremities, while muscle tone remained normal across all limbs. Tendon reflexes were absent, and Babinski’s sign was negative bilaterally. Examination of the cranial nerves and the autonomic nervous system yielded normal results.

The laboratory test results, as presented in **Table 1**, indicated a white blood cell count of 3.0×10^9/L, a 24-hour urinary protein excretion of 2458.8 mg, an antinuclear antibody titer of 1:1000, a positive result for anti-Smith antibodies, and a serum complement component C3 level of 0.67 g/L. Cerebrospinal fluid (CSF) analysis demonstrated a protein-cell dissociation phenomenon, characterized by a CSF nucleated cell count of 0 and a protein concentration of 777.2 mg/L. Electromyography and nerve conduction velocity assessments revealed peripheral nerve damage in all limbs, with pronounced demyelination and axonal degeneration in the distal extremities, predominantly affecting motor nerves. Computed tomography of the lungs indicated bilateral pleural effusion, while brain magnetic resonance imaging showed no abnormalities. Renal biopsy pathology, incorporating findings from immunofluorescence, electron microscopy, and light microscopy, was consistent with World Health Organization (WHO) class III lupus nephritis. The final diagnosis was systemic lupus erythematosus (SLE) complicated by Guillain-Barré syndrome (GBS).

**Table 1:** Laboratory tests.

Basic laboratory markers	Results	Reference range
Blood analysis		
CRP (c-reactive protein)	7.7	<10 mg/L
ESR (erythrocyte sedimentation rate)	68	0–15 mm/h
WBC (white blood cells)	3.0	3.5-9.5×109/L
Hb (hemoglobin)	128	130–175 g/L
PLT (platelet count)	248	125-350×10^9^/L
Urea	5.04	3.20-7.14 mmol/L
Creatinine	51.5	44.0-132.6 μmol/L
ALT (alanine Aminotransferase)	11.3	9–50 U/L
Alb (albumin)	35.9	40–55 g/L
ANA (antinuclear antibody)	1:1000	Negative
Anti-dsDNA antibody (anti-double-stranded deoxyribonucleic acid antibodies)	Negative	Negative
C3 complement	0.67	0.79-1.52 g/L
C4 complement	0.17	0.16-0.38 g/L
Anti-Sjogren’s SSA antibody	Positive	Negative
Anti-Sjogren’s SSB antibody	Negative	Negative
Anti-Ro52 antibody	Weakly positive	Negative
Anti-Smith antibody	Positive	Negative
Anti-U1RNP antibody	Positive	Negative
Anti-ribosome P protein antibody	Negative	Negative
Urine analysis		
Proteinuria	3+	Negative
24-hour Proteinuria	2458.8	<150 mg/24h

**Figure 2:**
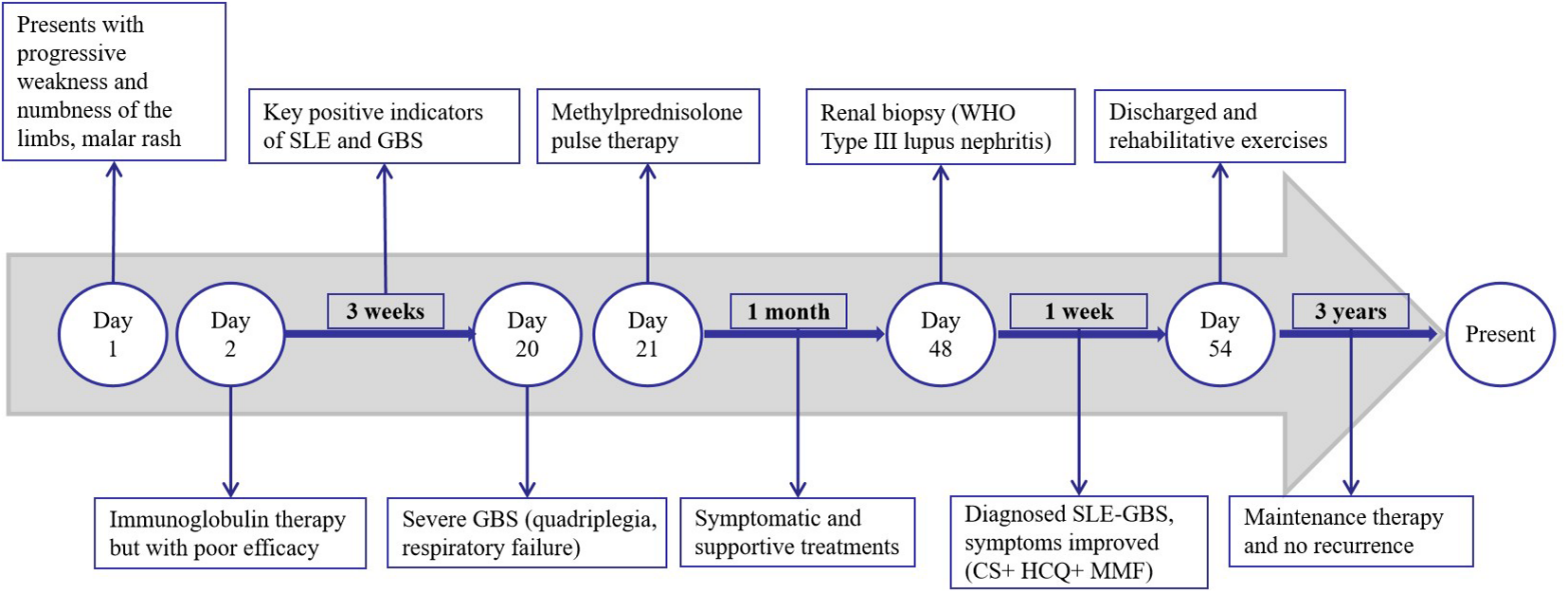
Sequential timeline of various events during the admission period.

The patient was initially administered intravenous immunoglobulin therapy (IVIg at a dosage of 0.4 g/kg/day for a body weight of 100 kg) over a period of five days; however, the response was suboptimal. Furthermore, the patient exhibited signs of autonomic nervous system dysfunction, including hypotension, urinary and fecal retention, and dyspepsia. Three weeks post-onset, the condition progressed to severe GBS, characterized by profound quadriplegia, dyspnea necessitating ventilatory support, and dysphagia requiring nasogastric feeding. Consequently, a regimen of methylprednisolone pulse therapy at a dosage of 1 g per day was administered for three days. Subsequently, the patient experienced a series of complications, including severe pneumonia, pulmonary embolism, and shock. Following one month of symptomatic management, which included ventilator-assisted ventilation, anti-infective therapy, and anticoagulation, the patient’s condition showed significant improvement. In the management of SLE, the patient received treatment with corticosteroids (CS), hydroxychloroquine (HCQ), and mycophenolate mofetil (MMF). Additionally, bedside neuroelectrophysiological monitoring was employed to inform and guide rehabilitation interventions. During hospitalization, there was a notable improvement in the patient’s malar rash and limb weakness. The post-discharge medication regimen included corticosteroids, initially administered at 70 mg/day and tapered by 15 mg monthly, eventually maintained at 10 mg/day for two years, and currently reduced to 5 mg/day. Hydroxychloroquine (HCQ) was continued at 0.1 g twice daily, and mycophenolate mofetil (MMF) was administered at 0.5 g twice daily, halved after one year, and discontinued following an additional year of maintenance. At the 9-month follow-up, muscle strength in all extremities had recovered to grade 5, although there was residual diminished pinprick sensation distally. Presently, the patient remains on 5 mg/day of corticosteroids and 0.1 g twice daily of HCQ, maintaining sustained remission without relapse over a three-year follow-up period. During the most recent outpatient visit, the patient expressed profound gratitude for the multidisciplinary medical care and familial support that facilitated their full recovery. A timeline of significant events during the patient’s admission is depicted in **Figure 2**.

## Discussion

SLE is a chronic autoimmune disease characterized by inflammation and immune-mediated injury to multiple organ systems, including the mucocutaneous, musculoskeletal, hematologic, and kidney systems (1). Globally, approximately 3.4 million individuals have been diagnosed of SLE, with a gender distribution of approximately one male to every nine females (3). In the Asia-Pacific region, the incidence rate is estimated to range from 2.5 to 9.9 per 100,000 annually, while the prevalence rate is approximately 3.2–97.5 per 100,000 annually (4). According to the 2019 EULAR/ACR classification criteria (3), the patient fulfills four clinical criteria (malar rash, leukopenia, pleural effusion, and lupus nephritis) and two immunological criteria (low complement C3 levels and a positive anti-Smith antibody) with a total score of 33, consistent with the diagnosis of SLE. The disease activity of SLE was evaluated by the SLEDAI (Systemic Lupus Erythematosus Disease Activity Index) 2000 criteria (5) with a total score of 11 (including proteinuria, rash, pleural effusion, hypocomplementemia, and leukopenia), indicating moderate activity.

GBS, characterized as an immune-mediated acute inflammatory peripheral neuropathy, typically reaches its peak clinical manifestation within approximately two weeks, and generally not exceeding four weeks (2). Most patients report antecedent events such as upper respiratory infection, diarrhea, vaccination, or surgical procedures. According to the Brighton criteria (6), the patient presented with symmetrical flaccid paralysis of the limbs, absent tendon reflexes, and a monophasic course (reaching a peak at 3 weeks). The cerebrospinal fluid showed protein-cellular dissociation, and electromyography revealed severe demyelination and axonal degeneration of the peripheral nerves in the limbs. Respiratory involvement was also observed. After excluding chronic inflammatory demyelinating polyneuropathy (monophasic course with no recurrence, electromyography results not supportive), spinal cord lesions (no sensory level or sphincter dysfunction), myasthenia gravis (no fluctuating symptoms, negative repetitive electrical stimulation test), and metabolic/toxic/infectious neuropathies (no corresponding medical history, serological and etiological tests negative), the diagnosis of acute inflammatory demyelinating polyneuropathy (AIDP) was confirmed.

Unlike many autoimmune disorders, GBS is more common in males than females and usually presents with a monophasic course, and only responds significantly to IVIG and PE. Therefore, GBS is not considered to be related to any chronic autoimmune disease (7). Based on the clinical characteristics of this case, we believe that the patient was SLE complicated by GBS. However, severe GBS manifestations are rare in similar reported cases. Severe GBS is characterized by rapidly progressing limb weakness, dysphagia, respiratory distress necessitating ventilatory support, and severe autonomic dysfunction (8). The primary causes of mortality in severe GBS are respiratory failure and cardiovascular complications, with a mortality rate reaching up to 20% among patients requiring mechanical ventilation (8). The patient in the present case exhibited rapidly progressing quadriparesis resulting in an inability to walk, dysphagia, respiratory failure, and autonomic dysfunction (including hypotension, urofecal disorder, and dyspepsia), aligning with the diagnosis of severe GBS. The occurrence of SLE complicated by severe GBS is rare, with severe manifestations involving respiratory failure being uncommon. Through a systematic literature review spanning cases reported since the seminal 1999 description of severe SLE-GBS with respiratory failure, we identified 13 severe cases (including the present case) meeting the above diagnostic criteria as of December 2024. Currently, a comprehensive characterization or comparative analysis of these cases remains conspicuously absent from the literature (9–20). We analyzed and summarized these thirteen cases, focusing on demographics, disease characteristics, diagnosis, treatment, and prognosis in **Table 2A**. Compared with classic GBS, severe SLE-GBS tends to occur at a younger age (average 33.6 years, compared with 50–70 years in classic GBS), a higher proportion of females (male-to-female ratio of 0.86:1, compared with 1.5:1 in classic GBS), no clear precipitating factors, a greater incidence of immune-related medical or family history, a higher rate of axonal damage, and a poorer prognosis (61.5% of severe cases fully recover, compared with 80% in classic GBS). Therefore the potential for SLE should be considered for patients with GBS without clear precipitating factors, young females, or those with immune-related past history or family history in clinical settings. The presence of axonal damage on electromyography further increases the likelihood of severe disease development, thereby complicating and heightening the risk associated with the patient’s condition. In this study, respiratory failure requiring mechanical ventilation was used as the screening criterion for severe cases of GBS (**Table 2B**). It should be noted that although rapidly progressive muscle weakness, severe swallowing disorders, and autonomic nervous system dysfunction are typical clinical manifestations of severe GBS, due to incomplete recording of relevant parameters in the case data, only respiratory failure was ultimately used as the inclusion criterion. This may have resulted in the exclusion of some patients who met the criteria for severe disease from the analysis.

**Table 2A:** Clinical and laboratory details of systemic lupus erythematosus complicated with severe Guillain-Barré Syndrome.

Citation ( Author / Time)	Age/ gender	Timing of GBS and SLE	Family history	Peak time	Induced factor	Cardinal symptom
Swati Vaidya (1999)	23/M	Concurrent	None	3 D	An upper respiratory tractinfection	Weakness, dysphagia, dyspnea
Eroboghene E. Ubogu (2001)	27/F	SLE first (2 months prior, maintenance treatment of CS)	None	19 D	Nonspecific abdominal discomfort without diarrhea	Weakness, paresthesia, intermittent diplopia and blurred vision in primary gaze
(11)	26/M	Mixed connective tissue disease or SLE with associated myositis first (5 years prior)	None	Not mentioned	None	Weakness, paresthesia, respiratory failure, dysphagia
YSantiago-Casas (12) (Case No.1)	20/F	Concurrent	None	Not mentioned	Fever (2 wk prior)	Weakness, respiratory failure, dysphagia, diplopia
Helen Chioma Okoh (2015)	41/F	Concurrent	None	6 D	A flu-like illness with diarrhea 5 months prior	Weakness, swelling, diplopia, blurry vision, respiration failure
Nan Zhang (14)	65/M	Concurrent	None	12 D	Fever, low back pain for 2 wk	Numbness, weakness, dysphagia, dyspnea
		(frheumatism for 40 yrs, HCV)				
Eric Anthony Coomes, (2018)	45/M	SLE first (2 years prior)	None	3 wk	None	Weakness, distal paraesthesia, ambulate inability, respiratory failure
Yamac Akgun, (2021)	20/F (pregnant)	SLE first (1 year without medications)	None	<3 wk	Flu-like symptoms 2 weeks prior	Numbness, weakness, facial diparesis with weak cheek puff and impaired eye closure
Elham Beshir (17)	14/F	Concurrent	Paternal family- hypothyroidism and G6PD deficiency	>4 wk	None	Paresthesia,diplopia, dysphagia
Amal Basnet (18)	22/F	Concurrent (fever for 6 months)	None	12 D	None	Paresthesia, weakness, respiratory failure
Samantha Braun (19)	34/F	GBS first (had malaria)	None	>5 D	None	Diplopia, weakness, paresthesias, neurogenic bladder, dysautonomia, neuropathic pain, respiratory failure
Jingqiao Wang (20) (Case No.3)	62/M	Concurrent	Not mentioned	1 wk	Not mentioned	Limb weakness, facial paralysis, respiratory and cardiac arrest
Present case	38/M	Concurrent	None	3 wk	None	Weakness, ambulate inability, respiratory failure

**Table 2B:** Clinical and laboratory details of systemic lupus erythematosus complicated by severe Guillain-Barré Syndrome.

GBS variant	Electromyogram results	Autonomic dysfunction	Cerebrospinal fluid analysis	SLE features
AIDP	Acute motor nerve demyelination	Not mentioned	Albuminocytologic dissociation	Fever, pleural effusions, leucopenia, complements, class V lupus nephritis
AMSAN	Severe axonal sensorimotor polyradiculoneuropathy with active denervation and sparing of the sural nerve	Not mentioned	Albuminocytologic dissociation (protein 55mg/dL)	Persistent fatigue, weight loss, malaise, arthralgias, nonerosive polyarthritis, oral ulcers, leucopenia, complements, class IV lupus nephritis
AIDP^a^	Axonal degeneration	Increasing abdominal distension	Albuminocytologic dissociation (protein 0.79 g/L)	Arthralgia, lymphadenopathy, myositis, necrotizing vasculitis, pericardial/pleural effusions, class I nephritis
AMAN	Acute motor axonal degeneration	Not mentioned	Normal	Fever, malaise, headache, lymphopenia
MFS	Prolonged distal onset latency, and severe conduction block	None	Normal ( WBC count 8/cumm; protein 35 mg/dL)	Pericardial/pleural effusions, leucopenia, complements, class III/V nephritis
AIDP	Severe demyelination	None	Albuminocytologic dissociation (WBC count 3.0×10^6^/L; protein 1.77 g/L)	Fever, low back pain, glomerulonephritis
AMSAN	Reductions in motor and sensory amplitudes without demyelinating features	None	Normal (WBC count 0×10^6^/L, protein 370mg/L)	Polyarthritis, pericarditis, pleuritis, hypocomplementaemia, class V lupus nephritis
CIDP				
AMAN	Acute motor axonal degeneration	Not mentioned	Albuminocytologic dissociation (protein 0.44 g/L)	Lupus nephritis
AMAN	Acute motor axonal degeneration	None	Albuminocytologic dissociation	Fever, left-sided knee and hip pain, class II lupus nephritis
AMSAN	Sensorimotor axonal polyneuropathy	Wide fluctuations in pulse and blood pressure and increased sweating	Albuminocytologic dissociation (absence of cells, protein 140 mg/dl)	Fever, malar rash, oral ulcers, photosensitivity, polyarthralgia
AMSAN	Diffuse motor axonal degeneration with new sensory involvement	Neurogenic	Normal	Fatigue, weight loss, night sweats, lymphadenopathy, class III lupus nephritis
		bladder, dysautonomia, neuropathic pain		
GBS	Not mentioned	Not mentioned	Albuminocytologic dissociation (protein 7.09 g/L)	Rash, proteinuria
AIDP^a^	Severe demyelination and axonal degeneration	Hypotension, urinary retention, dyspepsia	Albuminocytologic dissociation (WBC count 0×10^6^/L; protein 777.2 mg/L)	Malar rash,pleural effusion,leucopenia, class III lupus nephritis

The currently recommended treatments for GBS are plasma exchange and IVIg, while the definite efficacy of corticosteroids (CS) remains unconfirmed (21). However, in the specific clinical context of SLE comorbid with GBS, this treatment logic needs to be reexamined. According to current guidelines (22), when neuropsychiatric symptoms are highly correlated with SLE activity or specific neuropathies exhibit significant inflammatory features (such as axonal injury of peripheral nerves), high-dose corticosteroid pulse therapy (500-1000mg/d) should be employed as a core intervention strategy (15). Therefore, in the severe SLE-GBS cases presented in **Table 2C**, all patients received corticosteroid pulse therapy during the acute progression of the disease. Among them, five cases did not achieve significant efficacy despite early combination with IVIg, and their conditions were finally controlled through the addition of immunosuppressants such as cyclophosphamide and azathioprine. This phenomenon suggests that SLE complicated by GBS may have a unique pathological mechanism: compared to demyelinating lesions of peripheral nerves in classic GBS, SLE-GBS may involve a special mechanism dominated by complement activation/immune complex deposition. The combination of corticosteroids and immunosuppression can achieve acute phase control and reduce long-term risks by blocking T/B cell activation and cytokine storms, but this hypothesis urgently needs to be validated by multicenter randomized controlled studies.

**Table 2C:** Treatment and outcome details of systemic lupus erythematosus complicated by severe Guillain-Barré Syndrome.

Key positive indicators	Treatment	Outcome	Maintenance treatment	Other complication	Sequelae
Proteinuria, ESR, ANA (1:640), dsDNA (1:20), anti-Smith antibody, low C3,low C4	PE, CS (1g/d*3d), CP	Complete: the time of discharged.	CS	Pneumonia, worsening renal-function	None
ESR, ANA (1:1280), dsDNA (>400 IU), anti-Smith antibody, low C3, low C4, SSA	CS (1g/d*3d), CP, IVIG (improvement)	Complete: 7 months	CS	A herpes zoster infection, urinary tract infection	None
ANA (>1:1280), dsDNA(>1:160), RNP, Smith, anticardiolipin	CP, IVIG^b^, CS (60mg/d), PE	Complete: 10 D.	Died	Sepsis, multiorgan failure	Died
		*Recurred in 8 months, died			
Proteinuria, ANA (1:32), dsDNA	PE, IVIG^b^, CP, CS (60 mg/d*4wk), HCQ	Partial: 4 months	CS, HCQ	Not mentioned	None
		Complete: 10 years			
Proteinuria, ANA( 1 : (2560)), dsDNA(1:80), Smith>8,antiribosomal P antibodies >8, low C3,low C4 , GQ1	PE, Hemodialysis, CS (1g/d*3d), IVIG, CP	Complete: 18 wk	MMF, CS	Pancytopenia, pneumonia, sacral decubitus ulcer	No significant residual motor or sensory deficits
Proteinuria, ESR, CRP, dsDNA (1:10), low C3	IVIG^b^, CS(unknown)	Partial: 2 wk	Not mentioned	Not mentioned	Not mentioned
ANA, Smith, RNP, Ro	IVIG^b^, PE, CS(1g/d*3d+1mg/kg/d), MMF	Partial:several months	Rituximab, MMF, CS	Not mentioned	Slightly worse Strength and functional status
Proteinuria, ANA (1:640), dsDNA (98 IU/mL)	CS(1g/d*3d), IVIG^b^, CP	Complete: 6 months	Not mentioned	Not mentioned	None
Proteinuria, hematuria ESR,CRP, ANA (1:1280) dsDNA, Rheumatoid factor, low C3 , low C4 RNP, Smith, SSA , SSB	CS(1g/d*3d), IVIG^b^, rituximab, PE	Complete: 14 months	Rituximab, HCQ	Pneumonia	None
ANA(1:320),dsDNA, low C3, low C4	CS(1g/d*3d+1mg/kg/d), CP, IVIG, HCQ, PE	Complete: 3 months	CP	Not mentioned	None
Proteinuria, ribonucleoprotein extractable nuclear/anti-smith>8.0dsDNA 150 IU/mL	PE, IVIG, CS(unknown) , rituximab	Partial:5 months	HCQ, MMF, AZA	Not mentioned	Slightly worse Strength and functional status
Proteinuria (1.0 g/L), ANA (1:160), anti-ribP	IVIG, CS (80mg/d), intrathecal (dexamethasone 10 mg +methotrexate 10 mg times one cycle)	No marked improvement: not mentioned	Not mentioned	Not mentioned	Not mentioned
Proteinuria, ESR, ANA (1:1000), RNP, Smith, Ro52, SSA, U1RNP, low C3	IVIG^b^, CS (1g/d*3d), HCQ, MMF	Complete: 9 months	CS, HCQ, MMF	Pulmonary embolism, pneumonia, shock, hepatic insufficiency,intestinal dysfunction	A tingling sensation in some limbs

a The EMG suggested a secondary axonal injury.

b The IVIG treatment was lack improvement or worsen.

AIDP, acute inflammatory demyelinating polyneuropathy; AMAN, acute motor axonal neuropathy; AMSAN, acute motor and sensory axonal neuropathy; MFS, Miller-Fisher syndrome; AZA, azathioprine; CIDP, chronic idiopathic demyelinating polyradiculoneuropathy; Complements, hypocomplementaemia; CP, cyclophosphamide; CS, corticosteroid; IVIG, intravenous immunoglobulin; G6PD, glucose-6-phosphate dehydrogenase; MMF, mycophenolate mofetil; HCQ, hydroxychloroquine; HCV, hepatitis C virus; PE, plasma exchange; RNP, ribonucleoprotein; wk, week; yr, year; D, days.

This case represents a breakthrough in not adopting plasma exchange, and its successful experience highlights three core aspects: 1) Mechanical ventilation and hemodynamic monitoring during the peak period of respiratory muscle paralysis create a time window for neural repair; 2) Early bedside neuroelectrophysiological evaluation guides rehabilitation interventions to prevent disuse muscle atrophy; 3) A multidisciplinary team (with rheumatology leading immunomodulation, in collaboration with neurology, critical care, and rehabilitation departments) constructs individualized treatment pathways. This systematic treatment model suggests that SLE-GBS requires dynamic assessment of disease drivers based on guidelines, enhanced precision of immunosuppression, and synergistic effects of multidimensional supportive care.

## Conclusion

GBS is relatively prevalent in clinical settings. For female patients who manifest the disease at a young age, characterized by rapid progression, severe symptoms, unidentified precipitating factors, and a history of immune-related conditions or family history, the potential for SLE-GBS should be considered to facilitate early diagnosis. By examining a successfully diagnosed and treated case of severe SLE-GBS, we have synthesized the clinical characteristics of currently reported severe SLE-GBS cases and suggest the potential efficacy of moderate-to-high doses of glucocorticoids during the acute progression stage of SLE-GBS. This study provides a valuable reference for the diagnosis and treatment of such conditions.

## Data Availability

The original contributions presented in the study are included in the article/**Supplementary Material**. Further inquiries can be directed to the corresponding author.
