# Supplementary material for: Systemic lupus erythematosus complicated by severe Guillain-Barré syndrome: case report and literature review
Source: Front Immunol. 2025 May 9;16:1551448. doi: 10.3389/fimmu.2025.1551448 (PMC12098485; doi:10.3389/fimmu.2025.1551448)
Supplement: Supplementary file 1 [file DataSheet1.pdf]

CARE case report guidelines

CARE Checklist of information to include when writing a case report

(cc) BY-NC-ND

Topic	Item	Checklist item description	Reported on Line
Title	1	The diagnosis or intervention of primary focus followed by the words "case report"	1
Key Words	2	2 to 5 key words that identify diagnoses or interventions in this case report, including "case report"	7-8
Abstract (no references)	3a	Introduction: What is unique about this case and what does it add to the scientific literature?	18-19
	3b	Main symptoms and/or important clinical findings	15
	3c	The main diagnoses, therapeutic interventions, and outcomes	16
	3d	Conclusion—What is the main "take-away" lesson(s) from this case?	18-19
Introduction	4	One or two paragraphs summarizing why this case is unique (**may include references**)	32-34
Patient Information	5a	De-identified patient specific information.	37
	5b	Primary concerns and symptoms of the patient.	37-39
	5c	Medical, family, and psycho-social history including relevant genetic information	40-42
	5d	Relevant past interventions with outcomes	42-43
Clinical Findings	6	Describe significant physical examination (PE) and important clinical findings.	44-52
Timeline	7	Historical and current information from this episode of care organized as a timeline	88-89
Diagnostic Assessment	8a	Diagnostic testing (such as PE, laboratory testing, imaging, surveys).	53-64
	8b	Diagnostic challenges (such as access to testing, financial, or cultural)	106-110
	8c	Diagnosis (including other diagnoses considered)	110-115
	8d	Prognosis (such as staging in oncology) where applicable	76-79
Therapeutic Intervention	9a	Types of therapeutic intervention (such as pharmacologic, surgical, preventive, self-care)	75-79
	9b	Administration of therapeutic intervention (such as dosage, strength, duration)	79-87
	9c	Changes in therapeutic intervention (with rationale)	79-87
Follow-up and Outcomes	10a	Clinician and patient-assessed outcomes (if available)	
	10b	Important follow-up diagnostic and other test results	79-88
	10c	Intervention adherence and tolerability (How was this assessed?)	79-88
	10d	Adverse and unanticipated events	
Discussion	11a	A scientific discussion of the strengths AND limitations associated with this case report	144-148
	11b	Discussion of the relevant medical literature **with references**.	116-144
	11c	The scientific rationale for any conclusions (including assessment of possible causes)	149-164
	11d	The primary "take-away" lessons of this case report (without references) in a one paragraph conclusion	175-182
Patient Perspective	12	The patient should share their perspective in one to two paragraphs on the treatment(s) they received	87-88
Informed Consent	13	Did the patient give informed consent? Please provide if requested	Yes ☑ No ☐
